# Diagnosis of right bundle branch block: a concordance study

**DOI:** 10.1186/s12875-019-0946-3

**Published:** 2019-05-06

**Authors:** M. Alventosa-Zaidin, G. Pera, C. Roca Saumell, N. Mengual Miralles, M. V. Zamora Sanchez, T. Gros Garcia, L. Guix Font, M. Benitez Camps, J. Francisco-Pascual, J. Brugada Terradellas

**Affiliations:** 1 Bon Pastor, Primary Healthcare Center, Catalan Health Institute, Barcelona, Catalonia Spain; 2 Unitat de Suport a la Recerca Metropolitana Nord, Fundació Institut Universitari per a la recerca a l’Atenció Primària de Salut Jordi Gol i Gurina (IDIAPJGol), Mataró, Spain; 3 EAP El Clot, Primary Healthcare Center, Catalan Health Institute, University of Barcelona, Barcelona, Spain; 4 EAP Ronda Cerdanya, Primary Healthcare Center, Catalan Health Institute, Mataró, Barcelona Spain; 5 EAP El Gòtic, Primary Healthcare Center, Catalan Health Institute, Barcelona, Spain; 6 EAP Berga, Primary Healthcare Center, Catalan Health Institute, Berga, Barcelona Spain; 7 Unity of arithmies. Servei de cardiologia. University Hospital Vall Hebrón, Research Institut, Universitat Autònoma de Barcelona, CIBER-CV, Barcelona, Spain; 8 Cardiovascular Institute, Hospital Clínic, University of Barcelona, Catalonia, Spain

**Keywords:** Concordance, Bundle branch block

## Abstract

**Background:**

Right bundle branch block is one of the most common electrocardiographic abnormalities. Most cases of right bundle branch block are detected in asymptomatic patients in primary care, so a correct interpretation of electrocardiograms (ECGs) at this level is necessary.

The objective of this research is to determine the degree of concordance in the diagnosis of incomplete and complete right bundle branch block between four primary care researchers and a cardiologist.

**Methods:**

The research design is a retrospective cohort study of patients over 18 years of ages of patients over 18 years of ages who underwent an ECG for any reason and were diagnosed with right bundle branch block by their physician. The physicians participating, 4 primary care researchers and a cardiologist were specialized in interpreting electrocardiographic records. The diagnosis of incomplete and complete right bundle branch block was recorded and other secondary variables were analysed.

In case of diagnostic discordance between the researchers, the ECGs were reviewed by an expert cardiologist, who interpreted them, established the diagnosis and analysed the possible causes for the discrepancy.

**Results:**

We studied 160 patients diagnosed with right bundle branch block by their general practise. The patients had a mean age of 64.8 years and 54% of them were men. The concordance in the diagnosis of incomplete right bundle branch block showed a Fleiss’ kappa index (*k*) of 0.71 among the five researchers and of 0.85 among only the primary care researchers. The *k* for complete right bundle branch block was 0.93 among the five researchers and 0.96 among only the primary care researchers.

**Conclusion:**

The interobserver agreement in the diagnosis of right bundle branch block performed by physicians specialized in ECG interpretation (primary care physicians and a cardiologist) was very good. The variability was greater for the diagnosis of incomplete right bundle branch block.

## Background

Since the introduction of the Minnesota Code [1], several epidemiological studies have been published to determine the prevalence of ECG abnormalities in a standardized way. Most of these studies are based on the middle-aged population, mainly men in certain professions [2] .One of the abnormalities most commonly found is bundle branch block (BBB).

The heart’s natural pacemaker is the sinus node, which sends the electrical impulses to the atrioventricular node. From there the impulses are transmitted to the ventricles through the right and left branches of the His bundle (through Purkinje fibres). The ECG QRS complex indicates ventricular depolarization and under normal conditions is less than 120 ms.

When BBB occurs, one branch of the His bundle delays conducting the electrical impulse and the ventricle is activated by the myocardial propagation of the electrical activity of the other ventricle. Thus, the affected ventricle is depolarized erratically and slowly through an alternative pathway. This delay is shown in the ECG with a widening of the QRS complex (duration > 120 ms) and a change of its pattern, which varies depending on the affected branch.

One of the most frequent alterations of the ECG is Right bundle branch block (RBBB). [3]

Many studies have showed the association between RBBB with CV diseases [4] (cor pulmonale, myocarditis, acute myocardial infarction [AMI], pulmonary thromboembolism and congenital diseases) and this relation increases the CV morbidity and mortality. The new appearance of RBBB immediately after AMI therefore involves an increase in mortality [5]; and in patients hospitalized for exacerbated heart failure (HF) worsens their prognosis [6].

RBBB has been also associated with CVRF such as hypertension and diabetes mellitus [7, 8].

The impact of RBBB in patients with no history of CV disease is still controversial. Some studies have shown that RBBB increases CV events, with results not always statistically significant [9], or are only significant for a specific CV event [10, 11]. Whereas others studies have reported no risk [9, 12, 13].

There is no unanimous consensus on the diagnostic criteria of RBBB in the literature. All studies use a 12-lead standard ECG at rest, but there is no agreement on the wave abnormalities or their duration. Some studies use the diagnostic criteria of the Minnesota Code [10–16], but others and clinical practice guidelines use less stringent criteria [17–20].

The implications of detecting BBB, especially in prognosis, mean that ECG readings must be performed carefully for the conclusions to be valid. The articles cited above show great variation with regard to the correct interpretation of ECG by primary care physicians [21, 22].

After a thorough review of the literature, we found no studies that assess the degree of concordance of the diagnosis of RBBB between primary care (PC) physicians and cardiologists. Therefore, the aim of our study was to investigate the degree of concordance for the diagnosis of RBBB between 4 PC researchers and one cardiologist.

## Methods

The research design was a retrospective cohort study of patients over 18 years of ages. A concordance study was performed at an urban health centre in Barcelona. The ECG sample for the study was drawn from 3614 electrocardiographic records of 2147 adult patients in whom an ECG was recorded for any reason at the health centre during an 8-year period (2007 to 2015). Of the ECGs, 13.8% (*N* = 261) were interpreted by their PC physician as showing RBBB. Of these, we chose the ECGs that were well preserved to facilitate the interpretation of the researchers, resulting in a total of 160 ECGs. The remaining 101 ECG could not be used for being unreadable.

The 160 selected ECGs were given to the investigators of the project, who were not related to the centre where the ECGs had been recorded: 4PC physicians with special training in ECG interpretation and a cardiologist.

All researchers were unaware of the initial ECG diagnosis performed by the patient’s family physician, of the electrocardiographic record diagnosed by the other researchers, and of the automated analysis performed by the software.

### Assessment of RBBB in the ECGs

Before the researchers started to read the ECGs, the diagnostic criteria of complete RBBB (cRBBB) and incomplete RBBB (iRBBB) were agreed among all the participating researchers on the basis of the literature and clinical practice guidelines, in addition to the criteria of other abnormalities recorded: heart rhythm, heart rate, P wave duration, QRS duration, Cornell product, left bundle branch block (LBBB), left anterior and posterior hemiblock, atrioventricular block, bifascicular and trifascicular block, and atrial fibrillation.

**Fig. 1:**
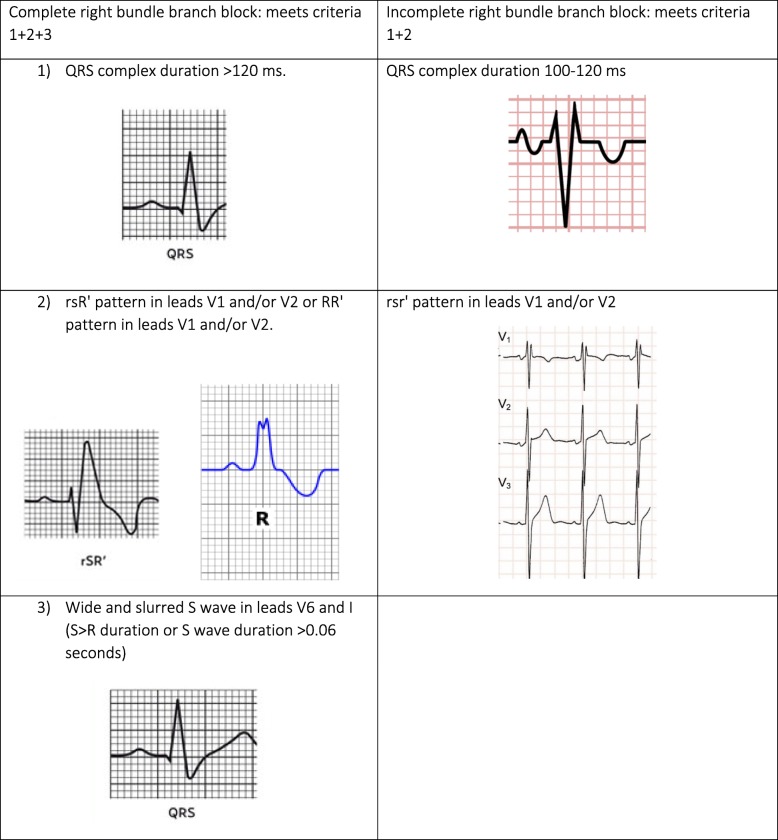
Diagnostic criteria for right bundle branch block^18,19^

Given the diversity of diagnostic criteria of RBBB, in the protocol the presence of cRBBB and iRBBB was defined using the most widely used criteria, which are shown in Fig. 1.

Patients with BBB with an rsr’ pattern in leads V1 and/or V2 but with a QRS duration of less than 100 ms were not labelled as having iRBBB, although in some studies they are considered as such. These patients were classified as patients with an rsr’ pattern but without diagnosis of iRBBB. The diagnostic criteria of other variables analysed are defined in Table 1.

**Table 1:** Secondary variables analysed

Variable	Description
Sinus rhythm	Regular PP interval Heart rate 60–100 bpm, P waves positive in lead II and negative in lead aVR PR interval constant (120–200 ms)
Electrocardiographic axis	Bayes de Luna criteria (34)
P wave duration, PR interval and QRS duration	In ms
Left ventricular hypertrophy (LVH)	Cornell index in mm: R in lead aVL + S in lead V3. Positive: > 28 mm in men > 20 mm in women
Left bundle branch block (LBBB)	M-shaped QRS complexes (RR’) in leads V5, V6, I and aVL. Wide and slurred S wave in lead V5 and/or V6. QRS complex duration > 120 ms: complete QRS complex duration 100–120 ms: incomplete
Left anterior hemiblock (LAH)	Marked left axis deviation ≥ − 30° Q1-SII-SIII pattern with wave SIII>SII. Typical rS pattern in leads II, III, aVF and qR in leads I, aVL.
Left posterior hemiblock (LPH)	Marked right axis deviation > 120° S1-QII-QIII pattern with wave RIII>RII. Normal QRS complex duration.
Bifascicular block	RBBB with LAH: RBBB pattern (QRS > 120 ms) + left axis deviation <−30°. RBBB with LPH: RBBB pattern (QRS > 120 ms) + right axis deviation <− 120°.
Trifascicular block	Bifascicular block plus first-degree AV block.
Atrioventricular block (AVB)	First-degree AVB: Constant PR interval with duration > 200 ms. Type I second-degree AVB: progressive lengthening of the PR interval until a beat is dropped. Type II second degree AVB: intermittent block of AV conduction without lengthening of the PR interval. Third-degree AVB: complete absence of conduction between the atria and the ventricles. P and QRS complexes follow an independent rhythm.
Cardiac arrhythmia due to atrial fibrillation (CAAF)	Absence of P waves Presence of irregular F waves (not always visible) Rapid frequencies and QRS complexes normal and arrhythmic (irregular PR interval).

The ECGs were interpreted individually by each researcher, who, after reading the electrocardiographic records, introduced in the database the presence or absence of RBBB, as well as the other parameters mentioned above. The data were later analysed centrally by a statistician who did not know who had interpreted the ECGs.

The ECGs that presented a diagnostic discordance (both cRBBB and iRBBB) between the five researchers (4 PC and the cardiologist) were reviewed by an expert cardiologist, who established the diagnosis and analysed the possible causes for the electrocardiographic misinterpretation.

### Statistical analysis

Each ECG was rated by 6 physicians (the 5 researchers and the PC physician who had performed the initial diagnosis). For the main variables (cRBBB or iRBBB), 6 diagnoses were obtained that could be coincident or not. The secondary variables were only analysed by the 5 medical researchers because we did not have the information from the patient’s family doctor. To establish the degree of concordance between the raters, for both the diagnosis of RBBB and the secondary variables analysed we used the Fleiss’ Kappa index (*k*) and its 95% confidence interval [23] For the continuous variables we used the interclass correlation coefficients (ICC).

For descriptive purposes, we present the box diagrams of the continuous variables evaluated by the 5 researchers. We used the Stata 14 statistical package for all the analyses.

## Results

Of the 160 patients with ECGs analysed, 54% were men. The mean age of the sample was 64.8 years (range, 18–97 years).

The results of the concordance between the PC physicians (who had made the initial diagnosis of RBBB) and the 5 investigators are shown in Table 2. The concordance between the diagnosis of the patients’ family doctors and the cardiologist was worse than that between the family doctors and the 4 PC researchers, though in both cases it was better for cRBBB than for iRBBB.

**Table 2:** Concordance between the PC physicians and the researchers

Variable	Degree of concordance (Fleiss’ kappa index and 95% CI)		
	PC physicians and the 5 researchers	PC physicians and the cardiologist	PC physicians and the 4 PC researchers
iRBBB	0.50 (0.46–0.54)	0.25 (0.13–0.37)	0.54 (0.49–0.59)
cRBBB	0.88 (0.84–0.92)	0.72 (0.57–0.87)	0.89 (0.84–0.94)

**Fig. 2:**
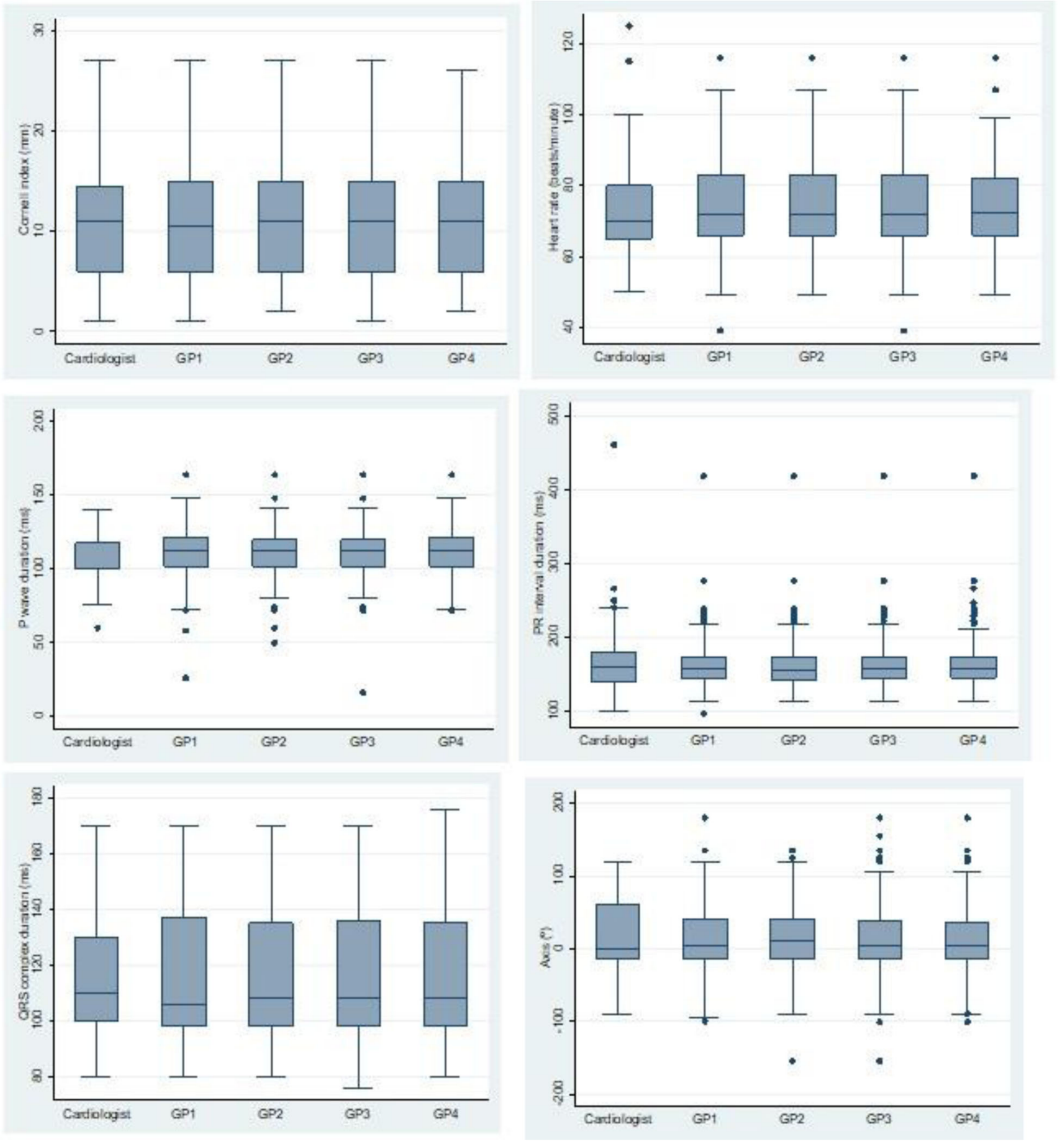
Box diagrams of Cornell index, heart rate, P wave, PR interval duration, QRS complex and axis, among the 5 researchers

The concordance for the ECG parameters between the 4 PC researchers and the cardiologist can be seen in Table 3. Again, the concordance was better for cRBBB and among the PC physicians. Regarding the continuous variables obtained from the ECGs, the ICCs were high, and they were higher between the 4 PC researchers than when the cardiologist was included. Figure 2 shows the box diagrams of these variables for the 5 researchers, with few differences between them.

**Table 3:** Concordance between the 4 PC researchers and the cardiologist and among the 4 PC researchers

Variable	Concordance between 4 PC researchers and the cardiologist.	Concordance between the four PC physicians
Primary variable		
iRBBB	0.71 (0.66–0.76)	0.85 (0.79–0.91)
cRBBB	0.93 (0.88–0.98)	0.96 (0.90–1.0)
Secondary variables		
Heart rhythm	0.75 (0.70–0.80)	0.77 (0.71–0.83)
Heart rate	0.96 (0.95–0.97)	0.98 (0.97–0.98)
Axis	0.86 (0.82–0.89)	0.89 (0.86–0.91)
P wave duration	0.70 (0.61–0.77)	0.84 (0.80–0.88)
QRS complex duration	0.96 (0.95–0.97)	0.98 (0.98–0.99)
PR interval duration	0.94 (0.93–0.96)	0.97 (0.96–0.98)
Cornell index: left ventricular hypertrophy (LVH)	0.88 (0.86–0.91)	0.89 (0.86–0.92)
Left anterior hemiblock (LAH)	0.82 (0.77–0.87)	0.82 (0.76–0.88)
Left posterior hemiblock (LPH)	0.24 (0.19–0.29)	0.16 (0.10–0.22)
Bifascicular block	0.79 (0.74–0.84)	0.81 (0.75–0.87)
Trifascicular block	0.74 (0.69–0.79)	0.76 (0.70–0.82)
Atrioventricular block (AVB)	0.69 (0.64–0.74)	0.73 (0.67–0.79)
Cardiac arrhythmia due to atrial fibrillation (CAAF)	0.82 (0.77–0.87)	0.84 (0.78–0.90)

Results are intraclass correlation coefficients (95% confidence interval)

Those ECGs that presented a diagnostic discordance (for both cRBBB and iRBBB) between the researchers were reviewed by an expert cardiologist, who established the diagnosis and analysed the possible causes of interpretation error.

There were 41 cases of discrepancy between the researchers in the iRBBB group. In 24 of them the discordance was between the cardiologist and the 4 PC researchers and in the remaining 17 ECGs it occurred between at least 2 researchers.

In the cRBBB group, there were 11 cases of discrepancy between the researchers. In 5 ECGs the different diagnosis was made between the cardiologist and the 4 PC researchers and, in the remaining 6, between at least 2 researchers. The description of the discordant ECGs and their causes are detailed in Table 4.

**Table 4:** Expert Cardiologist analysis of the discordant ECGs between the 5 researchers

cRBBB				
DISCORDANCE	1PC	3PC + iC	EC	Etiophatology
1	1 YES	1 NO	1 NO	QRS complex duration
DISCORDANCE	2PC	2PC + iC	EC	Etiophatology
2	2 YES	2 NO	2 NO	1 Axis 1 Morphology
3	3 NO	3 YES	2 NO 1 YES	2 Axis 1 QRS complex duration
DISCORDANCE	3PC	1PC + iC	EC	Etiophatology
-	–	–	–	–
DISCORDANCE	4PC	iC	EC	Etiophatology
4	4 NO	4 YES	4 YES	4 QRS duration
1	1 YES	1 NO	1 NO	1 Morphology
iRBBB				
DISCORDANCE	1PC	3PC + iC	EC	Etiophatology
8	8 YES	8 NO	1 YES 7 NO	1 Morphology 4 Morphology 3 Axis
2	2 NO	2 YES	2 NO	1 Axis 1 Morphology
DISCORDANCE	2PC	2PC + iC	EC	Etiophatology
6	6 NO	6 YES	1 YES 5 NO	1 Morphology 4 Morphology 1 Axis
1	1 YES	1 NO	1 NO	1 Morphology
DISCORDANCE	3PC	1PC + iC	EC	Etiophatology
-	–	–	–	–
DISCORDANCE	4PC	iC	EC	Etiophatology
14	14 YES	14 NO	9 YES 5 NO	13 Morphology 1 QRS complex duration
10	10 YES	10 NO	5 YES 5 NO	9 Morphology 1 Axis

*PC* Primary care researchers

*iC* Cardiologist who made initial diagnosis

*EC* Expert Cardiologist

## Discussion

### Summary

ECG is currently a routine clinical practice, not only in patients with clinical signs of myocardial damage (acute phases of cardiac pathology), but also in the general population for early detection of cardiovascular disease or risk factors. Clinical decisions following an ECG are dependent on the severity of the abnormalities found or the prognosis.

One of the most frequent abnormalities found is BBB, predominantly RBBB. RBBB is more frequent than LBBB because the Purkinje fibres of the right bundle branch have a longer, thinner structure than those of the left bundle branch. Therefore, minimal abnormalities in the Purkinje fibres, such as age-related degeneration, block the conduction of the right bundle branch [24].

### Strengths and limitations

Following the criteria of Landis and Koch [25] this study shows a level of concordance between good and very good among the PC researchers and the cardiologist for the diagnosis of RBBB. However, note that this criteria is arbitrary, although the closer k is to 1 the greater the agreement, specially if the number of categories is fixed, as it was. The concordance between the 5 researchers and the PC physicians who had made the initial diagnosis was worse than the concordance between the 5 researchers and the cardiologist.

Furthermore, for the secondary variables analysed, the concordance between the PC researchers and the cardiologist was also good or very good. However, this was not the case with concordance for posterior hemiblock, perhaps because the interpretation of the kappa index depends on the prevalence of the variable studied. In our study, only 3 cases were detected, one diagnosed by 3 researchers and the other 2 diagnosed by 2 researchers.

Most studies of the degree of competence of PC physicians in ECG interpretation are concordance studies between these physicians and cardiologists. This approach involves limitations, because there is also variability in the interpretation of the electrocardiographic abnormalities among cardiologists, and in the same cardiologist when an ECG is interpreted several times. This is a limiting factor in our study, so we also evaluated the degree of concordance among the 4 PC researchers but found no significant differences from the comparison with the cardiologist.

When we analyse the data of the ECGs with the electrocardiographic interpretation of the expert cardiologist, it is shown that, for the diagnosis of cRBBB, the expert cardiologist interpretation always matches the one made by the cardiologist on the initial analysis. For the iRBBB group, the expert cardiologist matches in some cases with the diagnosis of the PC researchers or with the initial cardiologist in others.

The main reason for the observed differences could be that the lecture of the ECGs was made manually, causing differences on the QRS complex duration, changes in the block morphology (the presence of r’ would not be clear) and, in some cases of discordance in iRBBB, the axis showed a left deviation and therefore the diagnosis was a left branch hemiblock.

Our study suggests that continuous education in ECG interpretation is a very effective tool for decreasing diagnostic variability and improving the competence of non-cardiologist physicians. However, we were unable to establish that it is the cause of the improved results, because we did not evaluate the concordance between the PC researchers and the cardiologist before and after the training and because the level of training of the PC physicians who had performed the initial diagnosis of RBBB was unknown.

The concordance results may have been overestimated because the researchers were better informed merely from participating in the study and because of the effect of the study design. It should be taken into account that the researchers may have found it easier to know and have available diagnostic criteria for RBBB than physicians working in usual day-to-day practice in PC or hospital consultation. Furthermore, the fact that only the best- preserved ECGs were chosen may constitute a selection bias, since in daily practice the quality of the electrocardiographic records is very variable.

### Comparison with existing literature and knowledge

Studies evaluating the correct interpretation of ECGs by general practitioners when compared with the diagnoses performed by cardiologists have shown that correct results are obtained in 36 to 96% of cases [21, 22]. Many studies consider the diagnostic capacity for a particular pathology. Some studies have observed that non-specialists in cardiology identify between 87 and 100% of myocardial ischemia [26], between 72 and 94% correctly classify patients who are candidates to receive thrombolytic treatment [27], between 57 and 95% detect abnormalities in the ST interval [28], and 25% perform correct interpretations of the PR and QT intervals [29].

There are discrepancies in assessing the impact on morbidity and mortality secondary to misinterpretation or non-identification of ECG abnormalities. In a systematic review, Salerno et al. [30], reported that the amount of diagnostic errors is between 4 and 33% but the incidence of secondary adverse effects is less than 1%. Other studies warn of the lack of competence of non-cardiologist physicians for detecting lethal electrocardiographic abnormalities that lead to an increase in avoidable cardiac morbidity and mortality [31, 32]. Prospective studies are necessary to evaluate the effect of RBBB in patients with no underlying cardiovascular disease. In patients with a history of cardiovascular disease, the presence of RBBB worsens the prognosis, so failure to identify it may have consequences for the patients.

### Implications for practice

The treatment and prevention of cardiovascular disease is one of the priority objectives in the daily work of the PC physician. Most cases of RBBB are detected in asymptomatic patients in PC, so a correct interpretation of the electrocardiograms (ECG) at this level is necessary.

Many studies [5, 33] show that the occurrence of RBBB immediately after a myocardial infarction almost doubles the risk of death, and this risk is higher than that of concomitant LBBB. Also, the appearance of RBBB in patients with heart failure who previously did have it worsens their prognosis [34, 35]. The clinical significance of RBBB in a patient with no evidence of any known cardiovascular (CV) pathology is a source of controversy.

Prospective studies are needed to evaluate the impact of RBBB in patients without baseline cardiovascular disease. If it is concluded that RBBB increases cardiovascular morbidity and mortality in healthy patients, it will be necessary to have new guidelines for treating and monitoring these patients.

## Conclusions

Our study shows that interobserver agreement in the diagnosis of RBBB performed by physicians specialized in ECG interpretation (both PC physicians and cardiologists) is very good. The variability is greater for the diagnosis of iRBBB, since there is greater diversity in the diagnostic criteria regarding the duration of the QRS complex. Unifying the criteria would help achieve a better diagnosis so that additional examinations can be made when necessary.

## Abbreviations

AVB: Atrioventricular block
BBB: Bundle branch block
cRBBB: Complete right bundle brunch block
ECG: Electrocardiogram
ICC: Correlation coefficients
iRBBB: Incomplete right bundle brunch block
*k*: Kappa index
LAH: Left anterior hemiblock
LBBB: Left bundle branch block
LPH: Left posterior hemiblock
LVH: Left ventricular hypertrophy
PC: Primary care
RBBB: Right bundle brunch block
